# The ability of probiotics to ameliorate blood and gonad damage caused by copper toxicity in Nile tilapia (*Oreochromis niloticus*)

**DOI:** 10.14202/vetworld.2021.2964-2970

**Published:** 2021-11-25

**Authors:** Alfiah Hayati, Manikya Pramudya, Hari Soepriandono

**Affiliations:** Department of Biology, Faculty of Science and Technology, Universitas Airlangga, Campus C, Mulyorejo, Surabaya, Indonesia

**Keywords:** copper, fisheries, fresh water, hematology, probiotics, testis structure

## Abstract

**Background and Aim:**

Industrial waste, such as heavy metals, is a major source of water pollution; at high levels, such pollution can reduce river water quality to the extent that it becomes unsuitable for aquaculture of freshwater fish. This study aimed to focus on the effects of copper (Cu) exposure in Nile tilapia (*Oreochromis niloticus*) and specifically the ability of *Lactobacillu*s-based probiotics supplementation in fish feed to ameliorate damage to gonads and negative effects on red blood cells (RBCs), whole blood cells (WBCs), hematocrit (HCT) levels, hemoglobin (HGB) levels, and malondialdehyde (MDA) levels following such exposure.

**Materials and Methods:**

Thirty-two Nile tilapia fish were divided into eight groups: A negative control (without probiotics or Cu), a positive control (with probiotics but without Cu), three treatments with probiotics in feed, and one of three Cu concentrations (0.75, 1.50, or 3.00 mg/L), and three treatments with these three Cu concentrations but without probiotics in feed. The probiotics concentration in feed was 25 mL/kg (1×10^8^ CFU/mL). Feeding was for 15 days, after which the hematological parameters, gonadal (testis) structure, and MDA levels of fish were analyzed.

**Results:**

Exposure to Cu significantly (p<0.05) affected fish hematology (decreased HGB, HCT, RBC, and WBC levels) and altered the structure of the testes. However, the addition of probiotics to fish feed significantly (p<0.05) ameliorated these effects on hematology and maintained the histological structure of the gonads.

**Conclusion:**

Cu exposure at ≥1.5 mg/L affected the hematologic parameters, gonadosomatic index, MDA levels, and testicular cells and tissue of Nile tilapia. However, probiotics supplementation in fish feed helped ameliorate the negative effects of Cu on these parameters. Thus, the *Lactobacillu*s-based probiotics used in this study were apparently able to neutralize Cu toxicity in Nile tilapia.

## Introduction

Freshwater aquaculture is generally conducted in rivers, ponds, lakes, reservoirs, or other water sources. Household waste resulting from human activities is a major source of pollution to such water sources; indeed, such pollution can alter the quality of river water to the extent that it is not suitable for use in aquaculture. Industrial waste, including that containing heavy metals such as copper (Cu), imposes an additional burden on river water quality and poses serious health risks [1,2].

Because heavy metals are not easily degraded, they are typically excreted together with feces and urine by fish. Consequently, fish are often used as indicators of heavy metal pollution in aquatic ecosystems; furthermore, heavy metals can be bioaccumulated and biomagnified through the food chain [3]. Heavy metals can enter the bodies of fish through the gills, body surface, or digestive tract [4]. After absorption, the metal is transported through the bloodstream to the organs and tissues where it accumulates. Heavy metals in the blood can cause blood abnormalities, thrombotic lesions, and necrotic cells. They can potentially increase *reactive oxygen species* (ROS) in endothelial cells, which affects the survival of fish. Increased oxidation can also alter blood glucose levels, protein concentrations, and hematological parameters, thereby leading to increased mortality. Furthermore, lipid oxidation reduces membrane integrity and DNA oxidation, which results in DNA fragmentation. The accumulation of heavy metals in the gonads of fish causes necrosis of the epithelium of seminiferous tubules and reduces sperm viability [5,6].

The World Health Organization defines probiotics as living microorganisms that provide host health benefits when the quantity consumed is sufficient [7]. The use of probiotics to control disease and improve water quality is prevalent in the aquaculture industry. LAB can reportedly remove heavy metals [8] by binding to cationic heavy metals that depend on pH; the pH effect arises due to competition for negatively charged binding between cationic metals and protons. Heavy metals are also removed due to microbes producing extracellular molecules known as siderophores, which have a high affinity for iron and can form complexes with other metals, even those with lower affinities. Siderophore–metal complexes can reduce metal concentrations and produce biosurfactant compounds that increase the solubility of heavy metals and thereby reduce their toxicity to cells [9].

Nile tilapias (*Oreochromis niloticus*) are sensitive to environmental changes and have been used as bioindicators of toxicity parameters [10]. In Indonesia, many studies have been conducted to analyze the effects of heavy metals on the physiology of Nile tilapia. However, few studies have reported the effects of probiotics on fish exposed to heavy metals, especially those exposed to Cu. Therefore, this study aimed to analyze the ability of probiotics supplementation in fish feed to ameliorate Cu exposure-induced gonad damage and the negative effects of Cu on the red blood cells (RBCs), whole blood cells (WBCs), hematocrit (HCT) levels, hemoglobin (HGB) levels, and malondialdehyde (MDA) levels of Nile tilapia.

## Materials and Methods

### Ethical approval

All procedures involving animal care were performed according to internationally recognized guidelines for the ethical use of animals [11] and Animal Care and Use Committee of Veterinary Faculty, Universitas Airlangga, Surabaya, Indonesia. Before being sacrificed, fish were made unconscious in 0.1 mL/L of clove oil containing eugenol. Clove oil solution was used to soothe the fish for several minutes without rejection reaction in fish [11].

### Study period and location

The study was conducted from May 2019 to March 2020. EThe experimental study was conducted in Animal Laboratory, Department of Biology, Faculty of Science and Technology, Universitas Airlangga.

### Probiotics for fish diet

The probiotics used in this study consisted of *Lactobacillus buchneri* (DSM 20057), *Lactobacillus casei* (DSM 20011), *Lactobacillus bulgaricus (NBRC13953)*, and *Lactobacillus fermentum* (ME3) in an equal ratio of 1:1:1:1. Each bacterial strain was cultured in de Man, Rosaga, and Sharpe broth for 24 h at 37°C. Subsequently, the culture broth from the four strains was mixed and centrifuged at 8000 *g* and 4°C for 15 min to precipitate the cells. Bacterial cells were then diluted using phosphate-buffered saline (PBS, pH 7.4) at a final concentration of 1×10^8^ CFU/mL. This concentration of probiotics was sprayed at approximately 25 mL/kg onto fish feed, which was then allowed air dry.

### Animals

Thirty-two male Nile tilapia (age: 4–5 months) in a healthy condition (weight: 200±20 g) with *mature* gonads was obtained from Pandaan Aquaculture Management Unit, Ministry of Fisheries, East Java Province, Indonesia. The fish were initially fed commercial pellets (30% protein, 3% fat, and 4% fiber; Takari, Sidoarjo, Indonesia) for 2 weeks of acclimation and kept in a glass rectangular tank (90 cm×60 cm×50 cm) utilized for fish-rearing. A 12-h light and 12-h dark photoperiod was used, and the pH range of the water was 7.5±0.03. An aquarium pump (Aquatic Pump Type WP-4880; 45 W) was positioned inside each rearing tank at the corner to recycle the water [12]. The freshwater was aerated at 25°C±1.5°C for 2 weeks of acclimation. The experimental feeding period was 15 days. During this period, the fish were fed with the experimental diets at 2% of their body weight twice daily at 7:00 am and 4:00 pm.

### Animal experiment

The 32 Nile tilapia was divided into eight groups: Two control groups, a negative control without probiotics or Cu and a positive control with probiotics but without Cu (negative control fish were kept in Cu-free water and fed the basal diet; positive control fish were kept in Cu-free water and fed the probiotics-supplemented diet), and six treatment groups exposed to Cu at one of three concentrations (0.75, 1.50, and 3.00 mg/L) that were fed with either the basal diet or the probiotics-supplemented diet. The concentration range for Cu exposure was chosen based on the LC_50_ value for Cu reported in a previous study [13]. All treatments were administered for 15 days.

### Fish hematology

Blood (1.0–1.5 mL) was collected using a disposable syringe (3 mL) from the caudal artery of Nile tilapia and then transferred to a special tube containing potassium ethylenediaminetetraacetic acid. At no more than 3 h after the blood sample was taken, hematological parameters were measured. Coulter counters (CELL DYN 1700) were used to calculate WBC, RBC, HGB, and HCT levels.

### Gonadosomatic index (GSI) estimation

An electronic balance was used to determine the total weight (g) of each fish specimen. The gonads were dissected and also weighed (g) to the nearest 0.01 mg. The GSI was calculated using the following formula:

        GSI = Weight of gonad×100

         Weight of fish.

### Cu determination in the gonads

Fish samples were rinsed with distilled water and dissected to separate the gonad using stainless steel instrument. 1One g of sample was crushed with perchloric acid and nitric acid with 1:1 in ratio, followed by sulfuric acid. The mixture was heated at 200ºC for 30 minutes. Then, it was cooled to room temperature (25-28ºC) and made to a scale of 50 mL with distilled water. Cu level was analyzed using ZEEnit 700 P atomic absorption spectrophotometer (AAS; flame and graphite furnace AAS systems, Analytik Jena, Germany) equipped with deuterium and Zeeman background correction, respectively, as recommended by the manufacturer. Detection limits were 0.046 μg/L for the flame AAS and 0.002 μg/L for the graphite furnace AAS.

### Determination of MDA in the gonads

Small pieces of gonad suspension were added to 300 mL of ice-cold PBS and homogenized on ice for 20 s using sonication. MDA concentration was then analyzed using a QuantiChrom TBARS Assay Kit (DTBA-100; BioAssay Systems, USA). The absorbance was measured at 535 nm.

### Histology of the gonads

Testes were fixed in Bouin’s solution for 24 h. The tissues were then dehydrated in an ascending series of alcohol, cleared in xylene, and finally embedded in paraffin wax. Sections (4-6 μm thick) were cut, processed, and stained with hematoxylin and eosin. They were examined under a light microscope (Olympus, Japan) and photographed using a built-in camera.

### Statistical analysis

All data related to hematological parameters and metal concentration levels are given as means±standard error. These data were tested using a factorial 2×4 (two levels of antibiotics and two levels of Cu) analysis of variance through the statistical package for the social sciences software package (IBM, United StatesNY, USA). Before this analysis, the normality and homogeneity of the data were confirmed. Means were subsequently compared using Fisher’s protected least significant difference test. Values were considered statistically significant at p<0.05.

## Results

### Hematological parameters

Table-1 shows the effects of Cu concentrations on the HBG levels of fish with and without probiotic supplements. At all Cu exposures (0.75, 1.50, and 3.00 mg/L), either with or without probiotics, HGB levels were significantly decreased comparedd to the Cu-free controls (p<0.005). However, probiotics supplementation in the positive control group increased HGB levels compared with those in the negative control group (from 9.0±0.2 to 10.8±1.0 g/dL). Similarly, all Cu exposure treatments showed a significant increase in HGB with probiotics supplementation (p<0.05). In contrast, there was no significant difference (p>0.05) among HGB levels in Cu exposure groups across concentrations in groups with or without probiotics supplementation.

**Table-1:** Effect of CuSO_4_ on hematology of *O. niloticus* with and without probiotic supplements.

Treatments (CuSO_4_ mg/L)	Probiotic (1×10^8^ CFU/mL)	HBG (g/dL)	HCT (%)	RBC (×10^6^/mm^3^)	WBC (×10^3^/mm^3^)
0	No	9±0.2^a^	27.5±0.70^a^	2±0.05^a^	137±7^a^
0.75	No	7.8±0.4^b^	22.2±0.80^b^	2±0.03^a^	122±5^b^
1.5	No	7.4±0.3^b^	20.2±1.20^b^	1.9±0.03^b^	120±2^b^
3	No	8.5±0.2^b^	24.7±0.50^c^	1.9±0.03^b^	127±1^b^
0	Yes	10.8±1.0^c^	31±2.00^d^	2.4±0.40^a^	129±3^a^
0.75	Yes	9.3±0.6^a^	28±1.50^ad^	2.1±0.10^a^	131±4^a^
1.5	Yes	9.3±0.6^a^	29±2.83^a^	2.1±0.03^a^	113±4^b^
3	Yes	9.9±0.2^a^	30±0.28^a^	2.1±0.03^a^	118±1^b^

RBC=Red blood cells, WBC=Whole blood cells, HCT=Hematocrit.

Different letters (a, b, c) showed a significant difference at p<0.05.

Probiotics supplementation also increased HCT levels; the negative control group had 27.5%±0.7% HCT, whereas the positive control group (with probiotics) had 31%±2.0% HCT. Cu exposure significantly reduced HCT levels with and without the probiotics supplement (p<0.05). However, the HCT percentage was higher in groups exposed to Cu concentrations and administrated probiotics than it was in Cu-exposed fish that were not administrated the probiotics supplement.

There was no significant difference between the RBC levels of the control group administrated with probiotics (2.4±0.4 10^6^/mm^3^) and those of the group not administrated with probiotics (2.0±0.05 10^6^/mm^3^). Exposure to 0.75 mg/L Cu did not affect RBC levels, but Cu at 1.50 and 3.00 mg/L reduced RBC count significantly (p<0.05). However, in these Cu-exposed groups, probiotic administration significantly (p<0.05) improved RBC levels.

Similarly, there was no significant difference (p>0.05) between the WBC levels of the control group administrated with probiotics (129±3 10^3^/mm^3^) and those of the group not administrated with probiotics (137±7 10^3^/mm^3^). However, the WBC count of fish decreased significantly (p<0.05) after exposure to Cu, probiotics supplementation could not significantly increase WBC counts in Cu-exposed fish at any Cu concentration (p>0.05).

### GSI analysis

The GSI of Nile tilapia exposed to Cu and those fed probiotics supplements are shown as index values in Table-2. The GSI of these fish was 1.08-1.33%. Without probiotics, there was no significant difference (p>0.05) in the GSI of fish exposed to 0.75 mg/L Cu and those in the control group, which shows that this Cu exposure level did not affect the gonadal development of the fish. However, the significant decreases (p<0.05) in GSI according to Cu concentration, 1.30%, 1.12%, and 1.08% for 0.75, 1.50, and 3.00 mg Cu/L, respectively, showed that higher Cu exposures could affect spermatogenic cell development. However, with the addition of probiotic supplements, the development of fish gonads was not affected in this way when they were exposed to the range of Cu concentrations. Indeed, probiotic supplementation significantly protected (p<0.05) the gonadal development of Cu-exposed fish, as shown by GSI values of 1.34-1.98%.

**Table-2:** Effect of CuSO4 on GSI, MDA, and heavy metal levels of Cu in the gonads of *O. niloticus* fish with and without probiotic supplements.

Treatments (CuSO_4_ mg/L)	Probiotic (1×10^8^ CFU/mL)	GSI (%)	MDA (μM/mL)	Cu levels in gonad (mg/L)
0	No	1.33±0.03^a^	1.40±0.01^a^	0.01±0.001^a^
0.75	No	1.30±0.08^a^	1.40±0.01^a^	0.39±0.004^b^
1.5	No	1.12±0.03^b^	1.47±0.02^b^	1.08±0.002^c^
3	No	1.08±0.02^b^	1.54±0.01^c^	2.46±0.03^d^
0	Yes	1.98±0.10^c^	1.38±0.01^d^	0.01±0.002^a^
0.75	Yes	1.8±0.20^c^	1.39±0.01^d^	0.30±0.03^b^
1.5	Yes	1.55±0.08^c^	1.38±0.01^d^	0.90±0.005^c^
3	Yes	1.34±0.10^a^	1.42±0.01^a^	2.33±0.001^d^

MDA=Malondialdehyde, GSI=Gonadosomatic index

Different letter (a, b, c, d) showed significant difference

### Heavy metal accumulation of Cu in fish gonads

In fish gonads, no significant difference (p>0.05) in Cu accumulation was observed in the negative and positive control groups. In addition, with the lowest Cu exposure (0.75 mg/L), there was no significant difference in Cu accumulation between the group administrated with probiotics and the group without probiotics. However, when Cu exposure was increased to 1.5 and 3 mg/L, the administration of probiotics significantly reduced the accumulation of Cu in the fish gonads (p<0.05; Table-2).

### MDA levels in the gonads

MDA levels in the fish gonads did not differ significantly (p>0.05) in the 0.75-mg/L Cu treatment groups. However, Cu exposure at 1.5 or 3 mg/L significantly (p<0.05) increased the level of MDA detected; thus, these exposures increased Cu oxidation stress. Nevertheless, the probiotics supplement was able to reduce MDA levels during exposure to different Cu concentrations. Thus, the probiotics apparently decreased oxidative stress caused by Cu (Table-2).

### Histological analysis

**Figure-1:**
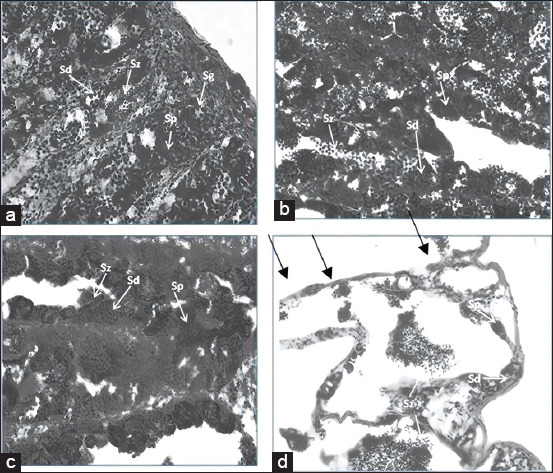
Levels of hemoglobin (a), percentage of hematocrit (b), red blood cells (c), and whole blood cell (d) exposed to heavy metals CuSO_4_. +p: with probiotics; −p: no-probiotics.

Male gonads in the control group showed a typical compact architecture with characteristic cyst distribution in the seminiferous tubules. Spermatogenic cell morphology at various stages of differentiation was noted as spermatogonium (Sg), spermatocytes (Sp), spermatids (Sd), and spermatozoa (Sz). Sg was found in cysts with various numbers of individual cells and was usually isolated near the periphery of the testes; cysts containing Sp, Sd, and Sz were located on the lobule wall (Figure-1a).

Sg and Sp were round with diameters of 49.5±4 and 39.8±5 µm, respectively. Sd were basophilic ball cells with a diameter of 25±3 µm; when they matured, they became smaller and remained in solid groups. Sz had a diameter of 16±3 µm (Table-3). Testicular histological examination of fish treated with increasing concentrations of Cu showed dose-dependent changes in the spermatogenic cell structure in cysts. Cu treatment induced complete disorganization in cyst settings. Several cysts were degenerated, which affected the development of spermatogenic cells (Figure-1b). Degeneration of cysts increased after exposure to 1.5 mg/L Cu (Figure-1c). Severe damage was observed with 3-mg/L Cu exposure as a reduction in the number of cysts and spermatogenic cells (Figure-1d). In addition, acute testicular atrophy and vacuolated seminiferous lobules were observed.

**Table-3:** The diameter of fish spermatogenic cells in control.

No.	Spermatogenic cells 5+p	Diameter (µm)
1	Spermatogonium	49.5±4
2	Spermatocytes	39.8±5
3	Spermatids	25±3
4	Spermatozoa	16±3

Probiotics supplements affected the spermatogenic cells in each cyst in the lobules. Cysts appeared to be the largest in the control group, as they were filled with spermatogenic cells (Figure-1a), followed by cysts in the group exposed to 0.75 mg/L Cu (Figure-1b). Cu exposure decreased the diameter of cysts; a major decrease was observed following exposure to 1.5 and 3 mg/L Cu (Figures-1c and d).

**Figure-2:**
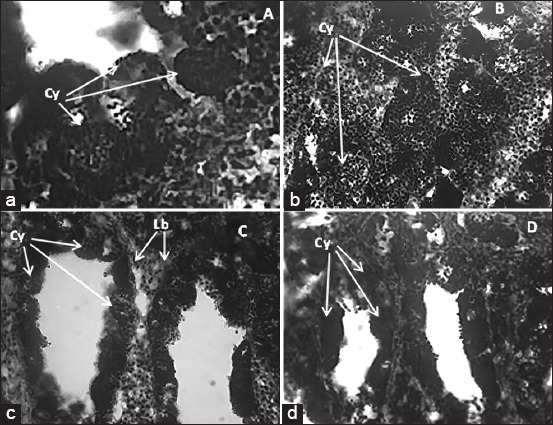
Photomicrographs of transverse sections of *Oreochromis niloticus* testes from various groups exposed to Cu with and without probiotic supplement. a=Control (0+p); b=0.75 mg/L Cu; c= 1.5 mg/L Cu; d= 3 mg/L Cu. Cy= cyst, Lb= Lobulus.

**Figure-3:**
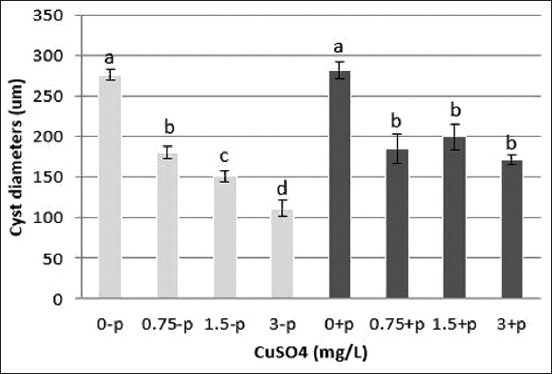
The diameter of cysts in the testicular lobule of *Oreochromis niloticus*. Values are represented as means±standard deviation (n=6). p<0.05 significantly different from control.

Spermatogenic cells protected in the cyst had various sizes depending on the treatment. The higher the Cu exposure level, the smaller the diameter (Figure-2). This was consistent with the structural reduction of the seminiferous tubules and cysts after exposure to Cu. Probiotics supplements were able to ameliorate the Cu-induced decrease in diameters (Figure-3). There was a significant difference (p<0.05) in the diameters measured among groups with and without probiotic supplementation.

## Discussion

Domestic waste, metallurgical activities, and industrial waste are all sources of anthropogenic metal input that result in the release of heavy metals into the environment [14]. A previous study revealed that the Brantas River in East Java contains high levels of heavy metal that exceed the government-determined threshold [2]. Many fish cultures are developed in freshwater, including rivers or reservoirs, for example, in freshwater fish cage cultivation [15]. Because fish are used primarily as food, it is necessary to consider heavy metal accumulation in aquaculture [16]. Our data showed that Cu was accumulated at high concentrations in the blood and testes of Nile tilapia, which can cause induction of lipid peroxidation and damage to cells and tissues.

We found that exposure to Cu significantly reduced the levels of HBG, HCT, RBCs, and WBCs in Nile tilapia (Figure-1). The decrease in these parameters may have been due to the destruction of mature RBCs and inhibition of erythrocyte production, as well as reduction in hemosynthesis that affects hematopathology or causes acute hemolytic crises that cause anemia in fish exposed to various environmental contaminants [17]. These results are in accordance with those of Srivastava and Punia [18], who found that toxic substances in water are a source of stress for aquatic organisms and that they cause physiological dysfunction, which induces changes in the blood parameters of fish, that is, significant reductions in RBCs and HCT indicative of anemia. Exposure to Cu also decreases WBCs, which indicates that Cu negatively affects the immune system of fish. WBC function is related to mechanisms that protect against the toxic reactions caused by heavy metal accumulation.

In the present study, increasing Cu exposure concentrations up to 1.5 mg/L significantly decreased the GSI in Nile tilapia (Figure-2). These effects show that cellular metabolism is affected by molecules that cannot be taken up by cells under heavy metal pressure [19]. Our study indicated that increased metal accumulation in fish testes is due to higher concentrations of metals in the water of their habitat. A major mechanism underlying heavy metal toxicity is oxidative stress, which arises from an imbalance between the production of free radicals and antioxidant defenses in organisms. Indeed, high concentrations of ROS are known to cause cell and tissue damage [10].

Here, the MDA levels of Nile tilapia increased as Cu exposure concentrations increased (Figure-4). Many studies have confirmed the presence of oxidative stress caused by ROS during heavy metal metabolism in cells [20]. ROS are considered to be directly involved in oxidative damage to lipids with the final product being MDA [21]. ROS accumulation disrupts the oxidant–antioxidant balance and causes cell damage and increased mortality [22].

ROS can also change the histological structure of fish gonads, which has been widely acknowledged in the previous work [5]. An abundance of evidence indicates that *in vivo* experimental exposure of adult male fish to heavy metals has detrimental effects on their gonad, gill, and liver histology, especially in *Barbodes* spp. [2]. In the present study, damage to the structure of the fish testicular tubules was caused by Cu exposure (Figure-1). Other studies have found that exposure to pollutants can cause a decrease in GSI [23], change the structure of testicular histology [2], reduce the quality of fish spermatozoa [6], and alter reproductive behavior [24].

Our data show that probiotic supplements can ameliorate the effects of Cu on hematological and GSI parameters (Figures-1 and 2), as well as decrease the absorption of Cu and MDA in the testes (Figures-3 and 4), and prevent Cu-induced histological damage to fish gonads (Figure-2). The protective mechanism of probiotics against heavy metal exposure generally involves binding to the heavy metal, inhibiting intestinal absorption, and protecting the intestinal barrier, which leads to reduced heavy metal accumulation, alleviation of oxidative stress, and mitigation of tissue damage [25]. Furthermore, *Lactobacillus* has the ability to export metals out of cells; thus, these bacteria can reduce cell and tissue damage to organisms by decreasing the cellular concentration of heavy metals. Bacterial cell walls act as a barrier against heavy metal ions; these ions bind to the peptidoglycan layer and teichoic and teichuronic acids found in cell walls. Therefore, the provision of probiotic supplements can protect against damage to fish cells and tissue during exposure to Cu.

## Conclusion

Cu exposure at ≥1.5 mg/L negatively affected the hematologic parameters and GSI of Nile tilapia while also increasing MDA levels as well as damage to testicular cells and tissue. However, probiotics supplementation in fish feed at a concentration of 25 mL/kg feed (1×10^8^ CFU/mL) helped protect HCT and HBG levels during Cu exposure. Probiotics also helped prevent damage to fish gonads during exposure to Cu. Therefore, probiotics based on the *Lactobacillus* group of bacteria were able to neutralize Cu toxicity in Nile tilapia.

## Authors’ Contributions

AH and MP: Participated in conceptualizing, designed experiments, and drafted the manuscript. AH, HS, and MP: Involved in experimentation, data collection, and data analysis. AH and MP: Involved in the preparation and revision of the manuscript. All authors read and approved the final manuscript.
